# Candidate Markers That Associate with Chemotherapy Resistance in Breast Cancer through the Study on Taxotere-Induced Damage to Tumor Microenvironment and Gene Expression Profiling of Carcinoma-Associated Fibroblasts (CAFs)

**DOI:** 10.1371/journal.pone.0070960

**Published:** 2013-08-08

**Authors:** Guohua Rong, Hua Kang, Yajun Wang, Tao Hai, Haichen Sun

**Affiliations:** 1 Department of General Surgery, Xuanwu Hospital, Capital Medical University, Beijing, P. R. China; 2 Surgery Lab, Xuanwu Hospital, Capital Medical University, Beijing, P. R. China; INRS, Canada

## Abstract

Recently, emerging evidence has suggested that carcinoma-associated fibroblasts (CAFs) could contribute to chemotherapy resistances in breast cancer treatment. The aim of this study is to compare the gene expression profiling of CAFs before and after chemotherapy and pick up candidate genes that might associate with chemotherapy resistance and could be used as predictors of treatment response. CAFs were cultured from surgically resected primary breast cancers and identified with immunohistochemistry (IHC) and Flow cytometry (FCM). MDA-MB-231 cells were cultured as the breast cancer cell line. Cell adhesion assay, invasion assay, and proliferation assay (MTT) were performed to compare the function of MDA-MB-231 cells co-cultured with CAFs and MDA-MB-231 cells without co-culture, after chemotherapy. Totally 6 pairs of CAFs were prepared for microarray analysis. Each pair of CAFs were obtained from the same patient and classified into two groups. One group was treated with Taxotere (regarded as after chemotherapy) while the other group was not processed with Taxotere (regarded as before chemotherapy). According to our study, the primary-cultured CAFs exhibited characteristic phenotype. After chemotherapy, MDA-MB-231 cells co-cultured with CAFs displayed increasing adhesion, invasiveness and proliferation abilities, compared with MDA-MB-231 cells without CAFs. Moreover, 35 differentially expressed genes (absolute fold change >2) were identified between CAFs after chemotherapy and before chemotherapy, including 17 up-regulated genes and 18 down-regulated genes. CXCL2, MMP1, IL8, RARRES1, FGF1, and CXCR7 were picked up as the candidate markers, of which the differential expression in CAFs before and after chemotherapy was confirmed. The results indicate the changes of gene expression in CAFs induced by Taxotere treatment and propose the candidate markers that possibly associate with chemotherapy resistance in breast cancer.

## Introduction

Breast cancer is considered as the most common cancer in women, accounting for 29% of estimated new cancer cases and 14% of estimated cancer-related deaths [1]. Chemotherapy is one of the cornerstone treatments in patients with breast cancer, which overall improves breast cancer outcome by 5–10% in patients with node negative disease [2]. However, its use is increasingly affected by chemotherapy resistance and lack of effective predictors. Recently, emerging evidences have suggested that carcinoma-associated fibroblasts (CAFs) could contribute to chemotherapy resistances in breast cancer treatment [3]. As the most frequent component of stroma cells in tumor microenvironment, CAFs have been assumed to play an important role in the carcinogenesis and development of breast cancer [4], [5]. Moreover, Farmer et al. reported that increased stromal gene expression predicts resistance to preoperative chemotherapy with 5-fluorouracil, epirubicin and cyclophosphamide (FEC), suggesting that stroma activation could be involved in chemotherapy resistance [6]. Additionally, it was shown that CAFs mediated resistance to chemotherapy by releasing collagen I [7]. Loeffler et al. have developed a vaccine that could target CAFs and allow reversing resistance to chemotherapy [8].

Considering the interaction between CAFs and chemotherapy resistance, it would be reasonable that chemotherapy-induced damage could have an impact on CAFs and change the expression of some relevant factors, which could participate in chemotherapy resistance. In our study, we have cultured CAFs which were derived from surgically resected primary breast cancers and compared the gene expression profiling of CAFs before and after chemotherapy. The goal will be to find candidate markers from tumor microenvironment that associate with breast cancer chemotherapy resistance and discuss the possibility that these markers could be used as predictors for chemotherapy efficiency and feasible for targeted therapy.

## Materials and Methods

### 1. Ethics Statement

The study was approved by the Institutional Review Board and Human Ethics Committee of Xuanwu Hospital, Capital Medical University. Written informed consent for using the samples for research purposes was obtained from all patients prior to surgery.

### 2. Cell Culture of CAFs and Breast Cancer Cell Line

Tissues for primary cultures of CAFs were collected from 10 breast cancer patients who underwent complete surgical resection of their tumors at Xuanwu Hospital, Capital Medical University. Only tissues in excess of those required for clinical diagnoses were harvested for this study. Harvested tissues were placed in DMEM supplemented with 10% FBS and antibiotics (Invitrogen Corporation) for immediate transportation on ice to the laboratory. Tissues were minced into small pieces, washed with phosphate-buffered saline (PBS) three times and digested for 20 h at 37°C in prepared reagent containing collagenase type I and Hyaluronidase (Roche Molecular Biochemicals). The cell suspension was filtrated with 100 mesh screen and centrifuged at 1000 rpm for 5 min, and then the pellet was resuspended in the fresh DMEM containing 10% FBS. Cell counting was performed with the Beckman Coulter Cell and Particle Counter Z1. The population doubling was estimated based on the increase in cell number counted at each passage time. Moreover, MDA-MB-231 cells were cultured in DMEM supplemented with 10% FBS as the breast cancer cell line, according to the normal procedure (Sigma-Aldrich).

### 3. Immunohistochemistry (IHC)

Primary antibodies for immunostaining included multi-cytokeratin (CK), Vimentin, α-smooth muscle actin (α-SMA), CD34, and TE-7 (anti-fibroblast antibody) (Labvision). CAFs were seeded in chamber slides and fixed in cold acetone. After antigen retrieval and blocking of endogenous peroxidase in 3% hydrogen peroxide, the cells were incubated with primary antibodies at room temperature in a moist chamber for 60 min. Specific signals were visualized by incubation with peroxidase-coupled secondary antibody for 60 min, followed by incubation with 3,3/-diaminobenzidine (DAB) used as a chromogen to create brown staining. Counterstaining was performed with hematoxylin for 5 min, and the slides were coverslipped.

### 4. Flow Cytometry (FCM)

CAFs were collected and prepared as a single cell suspension by mechanical blowing with PBS at the concentration of 1×10^5^/ml. The expression of CD34 and CD45 (MACS) was detected using FCM (FACSC alibar; BD).

### 5. Cell Adhesion Assay

The harvested MDA-MB-231 cells were diluted with DMEM at the concentration of 40000/ml, while CAFs were diluted at the concentration of 20000/ml. MDA-MB-231 cells were added to 24-well plates, which were divided into two groups. For one group based on co-culture assay, the filters were placed in 24 well plates and CAFs were added to each upper chamber (Transwell; Corning). For the other group, the filters and CAFs were not used. Both groups were then treated with 20 ng/ml Taxotere (Sanofi). Afterwards, the cells were incubated at 37°C in a humidified atmosphere of 5% CO_2_, until confluent. Matrigel (BD) was equilibrated with serum-free DMEM by proportion of 1∶3 before coating, and then 100 µl matrigel was added to each well in new 24-well plates. Two groups of MDA-MB-231 cells were harvested and transferred to 24-well plates coated with matrigel. After incubation for 1 h, MDA-MB-231 cells were washed with PBS, fixed in 4% formaldehyde and stained with 5% crystal violet. The number of MDA-MB-231 cells that adhered to the bottom of coated wells was counted and the morphology was recorded with an inverted microscope (Olympus IX70). The assay was done twice, each in triplicate.

### 6. Invasion Assay

The harvested MDA-MB-231 cells were diluted with DMEM at the concentration of 40000/ml, while CAFs were diluted at the concentration of 20000/ml. Matrigel was equilibrated with serum-free DMEM by proportion of 1∶3 before coating, and 50 µl/cm^2^ matrigel was added to each filter. The filters were placed in 24-well plates, which were divided into two groups. For one group based on co-culture assays, MDA-MB-231 cells were added to each upper chamber, and CAFs were added to the lower chamber. For the other group, the filters and CAFs were not used. Both groups were then treated with 20 ng/ml Taxotere and incubated at 37°C in a humidified 5% CO2 incubator for 72 h. At the end of the incubation period, the cells on the upper surface of the filters were removed with a cotton swab, and the filters were fixed in 4% formaldehyde and stained with 5% crystal violet. The number of cells that migrated to the lower side of the filter was counted and the morphology was recorded with an inverted microscope (Olympus IX70). The assay was done twice, each in triplicate.

### 7. Proliferation Assay (MTT)

The harvested MDA-MB-231 cells were diluted with DMEM at the concentration of 40000/ml, while CAFs were diluted at the concentration of 20000/ml. MDA-MB-231 cells were added to 24-well plates, which were divided into two groups. For one group based on co-culture assay, the filters were placed in 24 well plates and CAFs were added to each upper chamber. For the other group, the filters and CAFs were not used. Both groups were treated with 0 ng/ml, 4 ng/ml, 10 ng/ml, 20 ng/ml, 40 ng/ml Taxotere, respectively. Then the cells were incubated at 37°C in a humidified atmosphere of 5% CO_2_ for 72 h. Assays were initiated by adding 100 µl MTT (2 mg/ml) to each well and incubating the cells for an additional 4 h at 37°C. Afterwards, the medium was removed and 1 ml dimethylsulphoxide (DMSO) was added to each well. Finally, the supernatants were transferred to 96-well plates in triplicate, which were read at a wavelength of 550 nm with a Thermo Scientific Multiskan® Spectrum.

### 8. mRNA Expression Profiling

Totally 6 pairs of CAFs were prepared for microarray analysis. Each pair of CAFs were obtained from the same patient and classified into two groups. One group was treated with 20 ng/ml Taxotere for 24 h (regarded as after chemotherapy) while the other group was not processed with Taxotere (regarded as before chemotherapy). Total RNA was extracted from all cultured CAFs using the RNeasy kit (Qiagen) according to the manufacturer’s protocol. Microarray studies were performed by Capital Medical University Microarray Centre, using Illumina humanHT-12 v4 expression BeadChip based on Illumina BeadStation500. The biotinylated cRNA preparation, hybridization, and scanning of microarrays were done according to the manufacturer’s protocols. Biological replicates have been used to reduce errors. Illumina Gene Expression Beaderchips have internal control features to monitor data quality. The GenomeStudio software (Ilumina) calculates and reports a detection p-value, which determines whether a transcript on the array is called detected. In our study, a detection p-value below the threshold of 0.01 indicated that a gene could be considered as expressed. Differentially expressed genes in CAFs before chemotherapy vs. after chemotherapy were also identified and analyzed with GenomeStudio. The output was filtered to include genes whose expression was altered at least two-fold. The dataset of the microarray analysis has been deposited in ArrayExpress, with the accession number E-MTAB-1614.

### 9. Real-Time PCR

Real-Time PCR was performed to confirm differential gene expression in cultured CAFs before and after chemotherapy (treated with 20 ng/ml Taxotere for 24 h), using BIO-RAD IQ5 Real-Time PCR System. cDNA was synthesized using 1 µg total RNA, oligo (dT), and Superscript™ III Reverse transcriptase (Invitrogen). Synthesis was done according to the manufacturer’s instructions. All the primers were designed with Primer Express software (Applied Biosystems) for thecandidate genes. Predicted PCR product sequences were verified by using BLAST for recognition of target and non-target sequences.

### 10. Statistical Analysis

Statistical analysis was performed using SPSS 13.0 software (SPSS Inc). Student’s t test was used to test for statistical significance. Data were presented as the mean±standard error. *p*<0.05 was considered to indicate a statistically significant difference.

## Results

### 1. Characterization of Primary-cultured CAFs

**Figure 1:**
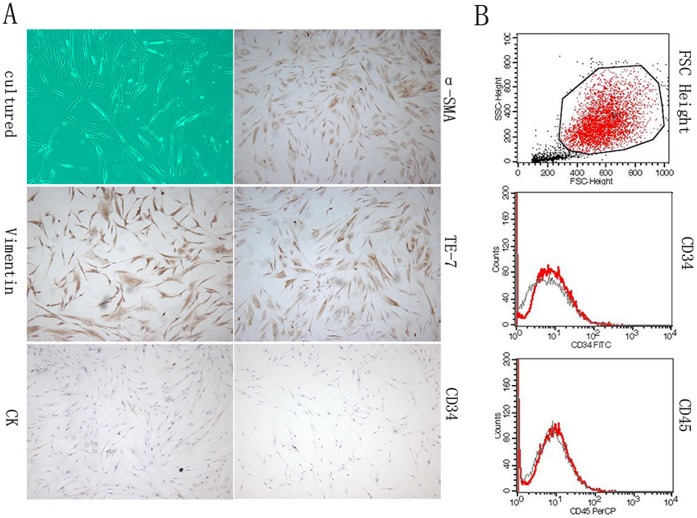
Characterization of primary-cultured CAFs. A showed the cultured cells which were morphologically characterized with flat spindle shape, rich cytoplasm and flat ovoid nuclear. With immunostaining, the primary-cultured CAFs exhibited positive expression of α-SMA, vimentin, and TE-7, but negative expression of CK and CD34. B showed negative expression of CD34 and CD45 in CAFs with FCM.

By using a study protocol approved by the Institutional Research Ethics Board, CAFs were cultured from 10 surgically resected primary breast cancers which were histologically confirmed. The cultured cells were morphologically characterized with flat spindle shape, rich cytoplasm and flat ovoid nuclear. With immunostaining, the primary-cultured CAFs showed positive expression of α-SMA, vimentin, and TE-7, but negative expression of CK and CD34. The morphological and immunohistochemical pictures of CAFs were represented in Figure 1A. Additionally, FCM showed negative expression of CD34 and CD45 in CAFs (Figure 1B).

### 2. CAFs Promotes the Function of Breast Cancer Cells after Chemotherapy

**Figure 2:**
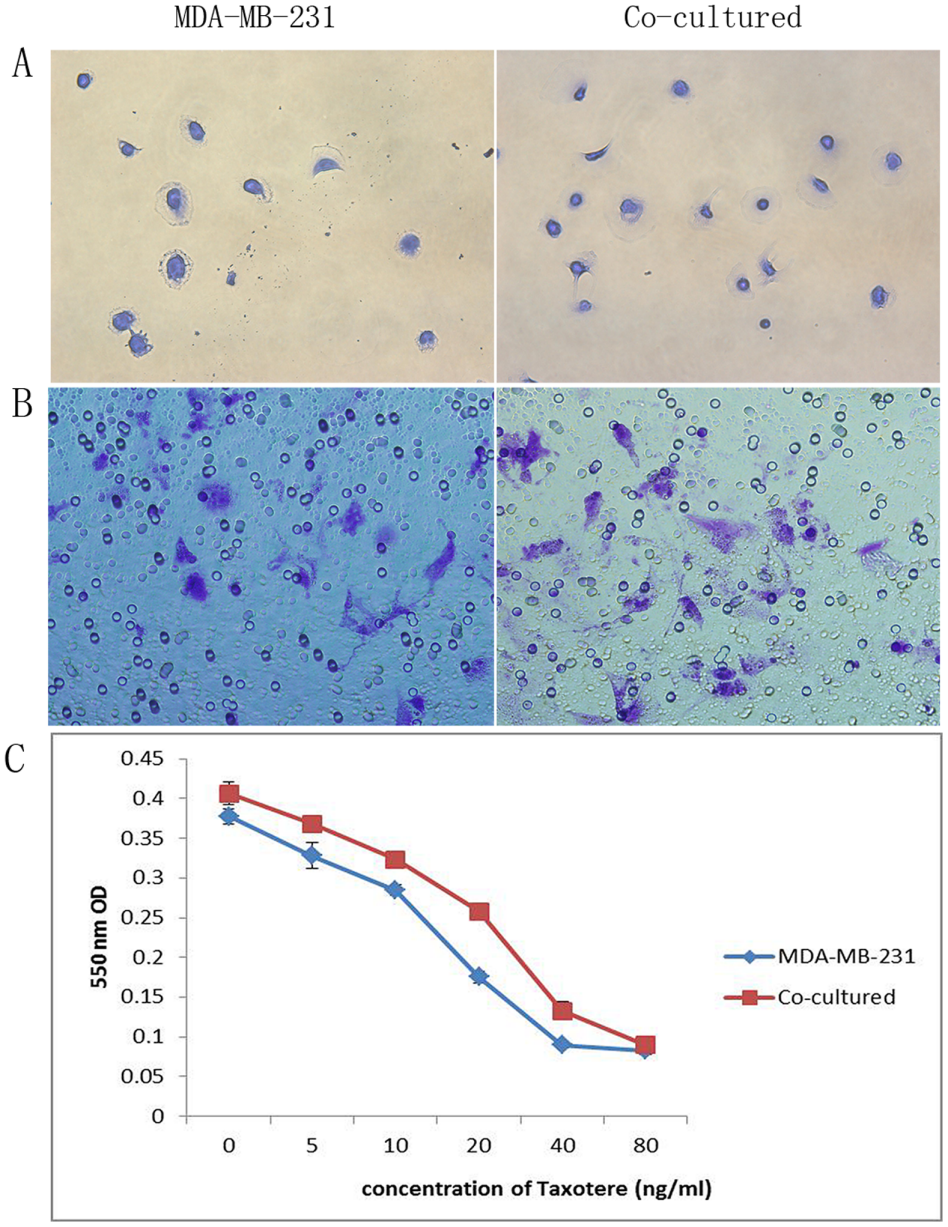
The function comparison of MDA-MB-231 cells co-cultured with CAFs and MDA-MB-231 cells without co-culture, when treated with Taxotere. By using cell adhesion assay (A), invasion assay (B), and MTT assay (C), it was observed that after chemotherapy, MDA-MB-231 cells co-cultured with CAFs displayed increasing adhesion, invasiveness and proliferation abilities, compared with cells without co-culture.

We assumed that chemotherapy-induced damage interacted with tumor microenvironment and hence compared the function of MDA-MB-231 cells after chemotherapy (treated with Taxotere) co-cultured with CAFs and that without CAFs. By using cell adhesion assay, invasion assay, and proliferation assay (MTT), it was observed that after chemotherapy, MDA-MB-231 cells co-cultured with CAFs displayed increasing adhesion, invasiveness and proliferation abilities, compared with MDA-MB-231 cells without CAFs. The representative pictures of cell functional studies were shown in Figure 2.

### 3. Comparison of Gene Expression Profiling in CAFs before and after Chemotherapy

**Figure 3:**
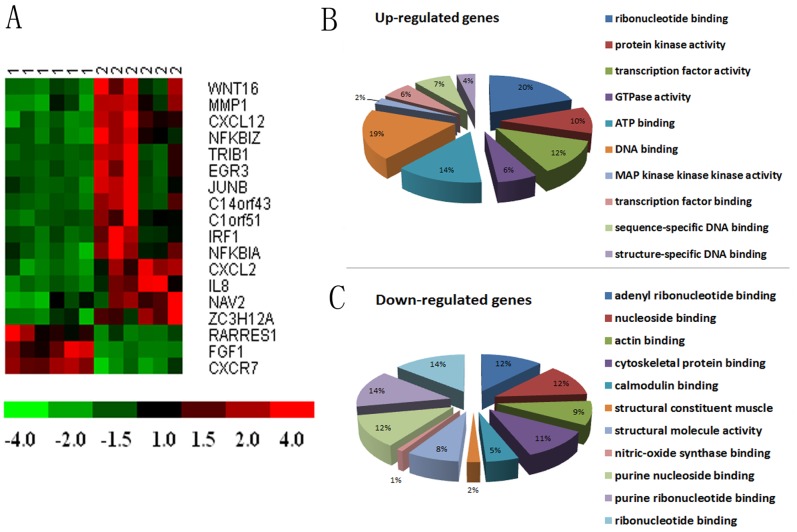
Hierarchical clustering of gene analysis expression and Gene Ontology (GO) analysis of CAFs before and after chemotherapy (treated with Taxotere). A showed the Heatmap plot of scaled gene-expression levels. The first 6 columns marked with “1” on top have represented the samples untreated with Taxotere, while the following 6 columns marked with “2” have represented the samples treated with Taxotere. Rows are genes with mean fold change between CAFs before and after chemotherapy. B and C revealed that these genes including up-regulated genes and down-regulated genes were mainly involved in nucleotide binding, actin binding, cytoskeletal protein binding and structural molecule activity through GO analysis.

Totally 24314 expressed genes were detected in the microarray assay and 35 differentially expressed genes were identified (absolute fold change >2) between CAFs after chemotherapy (treated with 20 ng/ml Taxotere) and before chemotherapy, including 17 up-regulated genes and 18 down-regulated genes. The differentially expressed genes were summarized in Table S1 and Figure 3A with clustering analysis. Moreover, Gene Ontology (GO) analysis revealed that these genes were mainly involved in nucleotide binding, actin binding, cytoskeletal protein binding and structural molecule activity (Figure 3B). The differentially expressed genes were also annotated in several Kyoto Encyclopedia of Genes and Genomes (KEGG) pathways, including focal adhesion (hsa04510), Regulation of actin cytoskeleton (hsa04810), and MAPK signaling pathway (hsa04010).

### 4. Differential Expression of Candidate Genes in CAFs before and after Chemotherapy

**Figure 4:**
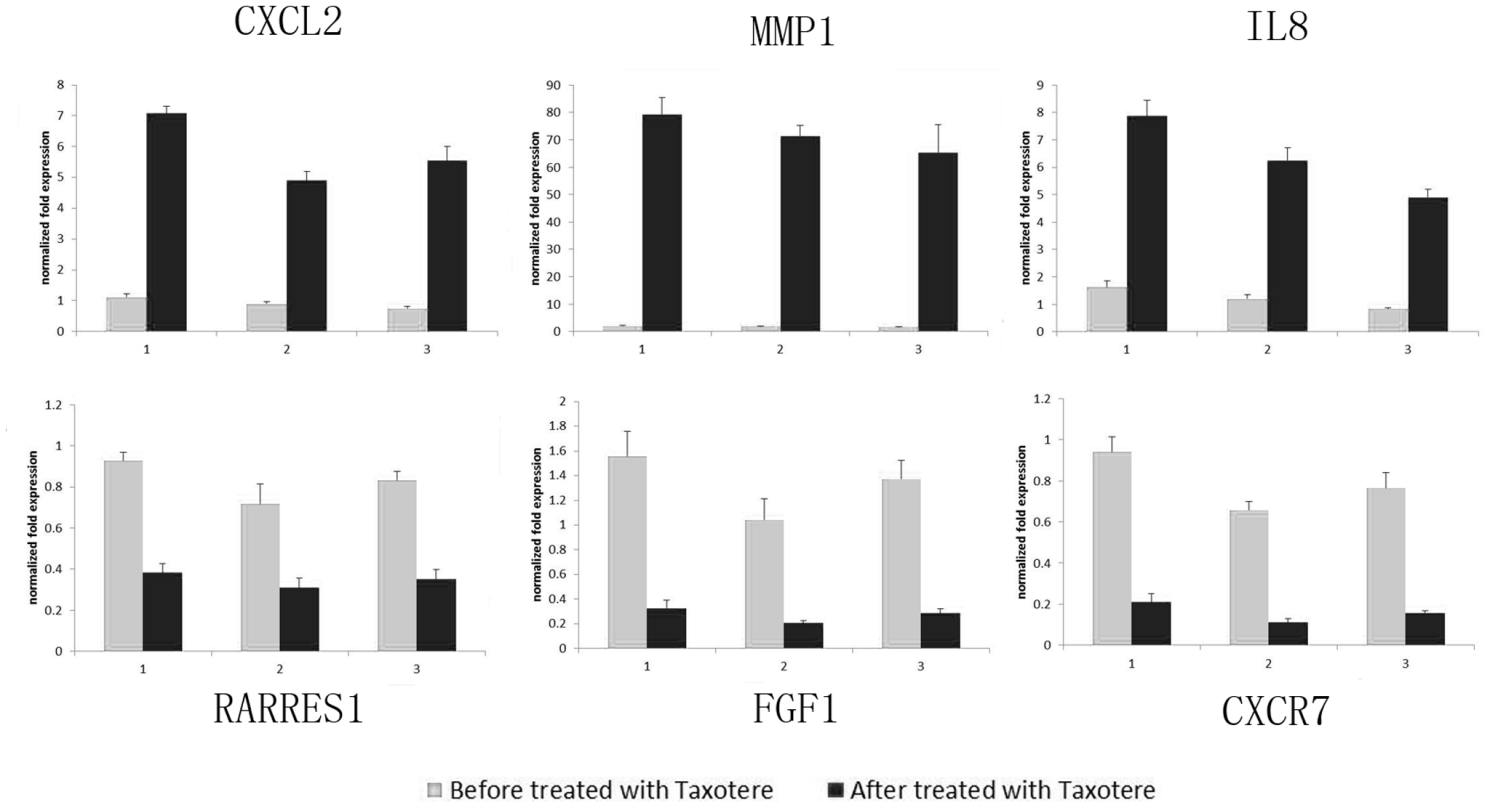
Differential expression of candidate genes in CAFs before and after chemotherapy. The candidate genes were picked up from 35 differential genes, including up-regulated genes CXCL2, MMP1, IL8, as well as down-regulated genes RARRES1, FGF1, and CXCR7. The differential expression of 6 genes in three pairs of CAFs before and after chemotherapy (treated with 20 ng/ml Taxotere) was confirmed by Real-Time PCR. It was found that there was significant difference between the expression of 6 candidate genes in CAFs before chemotherapy and after chemotherapy (*p*<0.05).

We have picked up 6 genes from 35 differential genes and confirmed the different gene expression in CAFs before and after chemotherapy (treated with 20 ng/ml Taxotere) via Real-Time PCR, using triplicate samples. The candidate genes included up-regulated genes CXCL2, MMP1, IL8, as well as down-regulated genes RARRES1, FGF1, and CXCR7. It was found that there was significant difference between the expression of 6 candidate genes in CAFs before chemotherapy and after chemotherapy (*p*<0.05). The pictures were represented in Figure 4.

## Discussion

As is known, cancer is a systemic disease encompassing multiple components of both tumor cells themselves and tumor microenvironment [9]. The notion is now widely accepted that the development and progression of cancer highly depends on the interactions between tumor cells and tumor microenvironment. Recently, many investigations have pointed to stromal cells as the major regulator in tumor initiation, progression, and metastasis of breast cancers [10], [11]. However, the origin of CAFs has been a debate. Based on different theories, CAFs might arise from activated resident fibroblasts, bone-marrow-derived mesenchymal stem cells, cancer cells that undergo epithelial-mesenchymal transition (EMT), or other undetermined mechanisms [12], [13], [14], [15]. Correspondingly, CAFs were reported to exhibit different expression of multiple biomarkers such α-SMA, FSP-1, FAP, platelet-derived growth factor-α receptor (PDGFR-α), platelet-derived growth factor-β receptor (PDGFR-β), vimentin, CAV-1, PTEN, p21, or TP53 mutation [16], [17], [18], [19], [20]. According to our study, CAFs showed positive expression of α-SMA, vimentin, TE-7 (anti-fibroblast antibody) and negative expression of CK, CD34, as well as CD45, suggesting that these primary-cultured CAFs were more likely to arise from activated resident fibroblasts, rather than epithelial cells, endothelial cells or bone marrow. We are aware that the observation based on morphological characteristics is just weak evidence for explaining the origin hypothesis, which needs further and more fundamental studies. Moreover, our data showed that CAFs could promote the adhesion, invasion and proliferation of breast cancer cells, which was consistent with peer researches.

Breast cancer is known as the leading cause of death in women worldwide. Neoadjuvant chemotherapy (NAC) has been considered as an effective way which could improve the outcomes especially in patients with advanced and inflammatory diseases. However, the resistance of tumor cells to a broad range of chemotherapeutic drugs and lack of useful predictive markers of response to NAC continue to be problems [21], [22], [23]. Though the precise nature and molecular mechanism of chemotherapy resistance is still unclear, many current studies have focused on identifying novel predictors of chemotherapy efficiency [24], [25]. CAFs merit attention, in consideration of frequent association with chemotherapy resistance. Sonnenberg et al. reported highly variable response to cytotoxic chemotherapy in CAFs from lung and breast, which could explain some levels of resistance in stroma-positive tumors where stroma would not be sensitive to chemotherapy [26]. Furthermore, an oral DNA vaccine targeting fibroblasts activation protein (FAP) was developed to suppress primary tumor cell growth and metastasis of multidrug-resistant murine breast carcinoma and allowed reversing resistance to chemotherapy, by increasing intratumoral drug uptake [8]. Based on our data, the comparison of gene expression profiling between CAFs before and after chemotherapy indicated the solid gene changes and provided candidate markers that might participate in chemotherapy resistance. Considering the correlation of breast cancer treatment and tumor microenvironment (especially CAFs), we suppose that the genes about membrane protein and secreted factors will be likely to associate with chemotherapy resistance. Then we have looked through the relevant literatures, to evaluate the research status and prospect of these genes, and eventually chosen CXCL2, MMP1, IL8, RARRES1, FGF1, and CXCR7 as candidate genes. The differential expression of these genes in CAFs before and after chemotherapy was confirmed by RT-RCR (p<0.05), suggesting potential predictors of response to treatment.

CXCL2 is one member of a family of structurally related chemokines, which are also called ELR-positive subgroup of CXC-chemokines [27]. It was reported that CXCL2 could enhance survival of primary chronic lymphocytic leukemia cells in vitro and differential expression of CXCL2 in colon cancer had impact on metastatic disease and survival [28], [29]. In addition, CXCL2 was found to show significantly different expression in 5-FU responder and nonresponder breast cancer cell lines [30], suggesting its relationship with chemotherapy response. Matrix metalloproteinase (MMP) 1 has been focused on, in view of the association between its five polymorphisms and lung cancer risk [31]. Moreover, the study carried out by Li et al. established the relationship between TP and MMPs in cancer cell invasion [32]. Recently, the up-regulation expression of MMP1 was observed during human triple negative breast cancer cell line progression to lymph node metastasis in a xenografted model in nude mice, indicating potential targets involved in the control of metastasis [33]. Interleukin-8 (IL-8) is a pro-inflammatory cytokine which was indicated to correlate with the growth and progression of tumors [34], [35]. Some interesting observations were made with regard to the prognostic role of baseline plasma IL8 protein levels in breast cancer patients treated with weekly docetaxel [36]. Besides, Snoussi et al. pointed out that the polymorphisms in IL-8 and its receptor CXCR2 are associated with increased breast cancer risk and disease progress, implying that IL-8 and CXCR2 might contribute to breast cancer pathogenesis and aggressiveness [37]. Lee et al. found that increased expression of IL-8 in the tumor microenvironment enhanced colon cancer growth and metastasis [38], which is very inspiring for our research. All the above markers including CXCL2, MMP1 and IL-8 were up-regulated based on our study, while FGF1, RARRES1 and CXCR7 as follows were down-regulated.

As is known, breast cancer cells overexpress fibroblast growth factor receptors. Fibroblast growth factor 1 (FGF1) was reported to be especially suitable as chemotherapeutic drug carrier in light of its biological activity [39]. Additionally, FGF1-gold nanoparticle conjugates targeting FGFR could efficiently decrease breast cancer cell viability [40], suggesting the possibility for targeted therapy. According to these data, it could be assumed that decreased expression of FGF1 might be involved in the chemotherapy resistance. Retinoic acid receptor responder 1 (RARRES1) is a retinoid regulated gene, which is accounted as a tumor suppress gene and lost in many cancer cells [41]. It has been demonstrated that the down-regulation of RARRES1 is related to tumor growth of colorectal cancer and nasopharyngeal carcinoma [42], [43]. The investigation on the role of RARRES1 in the chemotherapy resistance is still rare, therefore its decreased expression in CAFs after chemotherapy caused our attention. CXCR7, as well as CXCR4, have been known as the receptors of chemokine CXCL12. Liberman et al. considered that CXCR7 would elicit anti-tumorigenic functions, and may act as a regulator of CXCR4/CXCL12-mediated signaling in neuroblastoma [44]. Recently Hernandez et al. found that CXCR7 impaired invasion of breast cancer, in contrast to CXCR4 [45]. We propose that CXCR7 would contribute to the chemotherapy resistance in the condition of treatment-induced damage to the tumor microenvironment. Moreover, in the abovementioned candidate genes, CXCL2, MMP1 and IL8 are recognized as secretory-type genes, which possibly could be tested in serum in the form of genes or proteins. Its relationship with prognosis will be studied.

Overall, in this study we have primarily cultured CAFs, compared its gene expression profiling before and after chemotherapy, and picked up 6 candidate genes which are possibly associated with chemotherapy resistance in breast cancer. We hope that our study might supply the potential predictors for chemotherapy efficiency and possible targets for treatment, which could provide the patient with optimal therapeutic management and better prognosis. For further study, the molecular mechanism of these candidate markers will continue to be researched to elucidate their relationship with chemotherapy resistance.

## Supporting Information

Table S1 **The differentially expressed genes in CAFs after vs. before chemotherapy.**
(DOCX)
