# Supplementary material for: Candidate Markers That Associate with Chemotherapy Resistance in Breast Cancer through the Study on Taxotere-Induced Damage to Tumor Microenvironment and Gene Expression Profiling of Carcinoma-Associated Fibroblasts (CAFs)
Source: PLoS One. 2013 Aug 8;8(8):e70960. doi: 10.1371/journal.pone.0070960 (PMC3738633; doi:10.1371/journal.pone.0070960)
Supplement: Table S1 — The differentially expressed genes in CAFs after vs. before chemotherapy. (DOCX) [file pone.0070960.s001.docx]

**Table S1** The differentially expressed genes in CAFs after vs. before chemotherapy

| Gene Symbol | Gene Description | Regulation |
| --- | --- | --- |
| ACTA2 | actin, alpha 2, smooth muscle, aorta | down-regulated |
| ACTC1 | actin, alpha, cardiac muscle 1 | down-regulated |
| ACTG2 | actin, gamma 2, smooth muscle, enteric | down-regulated |
| ALDH1B1 | aldehyde dehydrogenase 1 family, member B1 | down-regulated |
| AMY1A | amylase, alpha 1A (salivary) | down-regulated |
| C5orf13 | chromosome 5 open reading frame 13 | down-regulated |
| CNN1 | calponin 1, basic, smooth muscle | down-regulated |
| CXCR7 | chemokine (C-X-C motif) receptor 7 | down-regulated |
| DDAH1 | dimethylarginine dimethylaminohydrolase 1 | down-regulated |
| FGF1 | fibroblast growth factor 1 (acidic) | down-regulated |
| PDLIM3 | PDZ and LIM domain 3 | down-regulated |
| MAMLD1 | mastermind-like domain containing 1 | down-regulated |
| MYH11 | myosin, heavy chain 11, smooth muscle | down-regulated |
| OXTR | oxytocin receptor | down-regulated |
| PDLIM5 | PDZ and LIM domain 5 | down-regulated |
| RARRES1 | retinoic acid receptor responder (tazarotene induced) 1 | down-regulated |
| SERPINA3 | serpin peptidase inhibitor, clade A (alpha-1 antiproteinase, antitrypsin), member 3 | down-regulated |
| TRIL | TLR4 interactor with leucine-rich | down-regulated |
| C14orf43 | chromosome 14 open reading frame 43 | up-regulated |
| C1orf51 | chromosome 1 open reading frame 51 | up-regulated |
| CXCL12 | chemokine (C-X-C motif) ligand 12 | up-regulated |
| CXCL2 | chemokine (C-X-C motif) ligand 2 | up-regulated |
| EGR2 | early growth response 2 | up-regulated |
| EGR3 | early growth response 3 | up-regulated |
| IER3 | immediate early response 3 | up-regulated |
| IL8 | interleukin 8 | up-regulated |
| IRF1 | interferon regulatory factor 1 | up-regulated |
| JUNB | jun B proto-oncogene | up-regulated |
| MMP1 | matrix metallopeptidase 1 | up-regulated |
| NAV2 | neuron navigator 2 | up-regulated |
| NFKBIA | nuclear factor of kappa light polypeptide gene enhancer in B-cells inhibitor, alpha | up-regulated |
| NFKBIZ | nuclear factor of kappa light polypeptide gene enhancer in B-cells inhibitor, zeta | up-regulated |
| TRIB1 | tribbles homolog 1 (Drosophila) | up-regulated |
| WNT16 | wingless-type MMTV integration site family, member 16 | up-regulated |
| ZC3H12A | zinc finger CCCH-type containing 12A | up-regulated |
